# On the Nature of Volition

**Published:** 1863-04

**Authors:** J. Lockhart Clarke


### MEDICAL CRITIC

AND

### PSYCHOLOGICAL

### JOURNAL.

APRIL, 1863.

### Art. I.—ON THE NATURE OF VOLITION.

By J. Lockart Clarke, F.R.S.

Part III.

(*Continued from* No. ix. *p.* 24.)

In my last article I entered into an analysis of the different forms of volition, and showed that in each case the process consists in the co-operation of two of the psychæal elements which together constitute our personal integrity; namely, the Intellectual and the Æsthetic; that the intellectual is the *regulative* and directive element of "the will;" while the æsthetic is the *dynamic* or *active* element, and consists of either a sensation, an appetite, or an emotion, which, when it reacts through the regulative element, and is directed by it to its appropriate and special object or end, assumes the form of a special desire to act for the attainment of that end. It was then seen to follow, that what are called "motives" *to* "the will," consist of our various sensations, appetites, and emotions, when subjected to the judgment of the understanding in deliberation, and balanced against each other in reference to good or evil: that one or the other of these "motives," therefore, always constitutes the dynamic or active element of "the will" itself; and that what particular "motive" shall constitute this element of "the will," on any given occasion, must depend on its superior influence over others that may, at the same time, be presented to, or excited by, the understanding, which constitutes the complemental or regulative element of "the will." The "will," therefore, as a peculiar power, comes into existence only at the time of acting, by the combination and co-operation of its constituent elements.*

It is only by such an analysis of the various forms of volition that we are able to expose the absurdities and inconsistencies which may be found in the speculations of many of the most distinguished philosophers, especially in modern times, on the nature and acts of "the will." Those who hold the doctrine of a special faculty, consider it as an unknown something, altogether different from the rest of our faculties; and neither subject, like them, to any laws of action, nor capable either of analysis or definition. With so vague or indefinite a notion of its nature, it is not surprising that the speculations of those who attempt to explain the operations of this lawless and mysterious faculty should issue in absurdity and contradiction. According to this doctrine, it is wholly independent of the understanding, although it is manifestly dependent on the guidance of intelligence. It is said to be independent of the senses, the appetites, and emotions, although every one of its acts implies the necessary precedence of some kind of desire or aversion, which, in conjunction with intelligence, those feelings respectively excite. That such are the qualities imputed to this hypothetical faculty, is sufficiently evident. Listen to the words of one of its advocates: "The will," he says, "is determined neither by present uneasiness, nor by the greatest apparent good, nor by the last dictate of the understanding, nor by anything else, but merely by itself. And thus in some cases," he continues, "the will determines itself in a very sovereign manner, because it will, without a reason borrowed from the understanding."† The same view appears to have been taken even by so profound and subtle a thinker as the celebrated Dr. Samuel Clarke. In his answer to the assertion of Collins, that the act of judging of propositions is concerned in volition, he says, "Without all dispute, the perception of ideas is no action at all;" and continues, "as if seeing a thing to be true or false was an action, or had anything to do with the will." Let us observe the train of thought by which this great and venerable man appears to have been led to this conclusion. By "action" he evidently meant, not intellection, but an external act producing movement. Now finding that the judgment of the understanding—especially the speculative judgment—has

* The above view of the nature and operations of "the will" sets at rest the long and hotly-debated question, whether, or how, "motives" act on or determine the "will." "The great question," says Dugald Stewart, "is, How do these motives determine the will?" And again, "The question is not concerning the influence of motives, but concerning the nature of that influence."—*Philosophy of the Active and Moral Powers.* Appendix, sect. iv. (vol. vi. p. 346, of Hamilton's Stewart.)

† *Essay on the Freedom of the Will.*

no immediate power of exciting a voluntary movement, he therefore concluded that the understanding is passive; and so in this sense it really is, although in its practical capacity it is one of the constituent elements, and therefore one of the essential conditions, of the will. "The last perception or judgment of the understanding," says Dr. Clarke, "is entirely passive." But the "first exertion of the self-moving power," he continues, "is essentially active." Therefore (he concluded) the understanding cannot be the self-moving power—that is, the free-will. But if the free-will is not the understanding, much less could it be supposed to consist of the mere brute faculty of sense or appetite. There was therefore invented a special self-moving faculty, which was supposed to be independent of all the senses, the appetites, and the understanding. The great error of Clarke, and of others who adopt the same opinion, consists in their not perceiving that the will is compounded essentially of two distinct elements, the active and the regulative, which must always combine and co-operate with each other in every actual volition.

But for want of a proper analysis and clear understanding of the actual process of volition, even many of those philosophers who profess to reject the doctrine of a special faculty, do virtually, by their reasonings or assertions, admit or imply its existence, with all its consequent absurdities. Let us see what an eminent metaphysician, Mr. Mansel, has to say on the subject. In a brief and general definition of the *will*, he denies its claim to rank as a special faculty. "Will, like sense and reason," he says, "does not indicate a special phenomenon of consciousness, but is a general name for the power from which special phenomena proceed."* We shall find, however, that in claiming for the *will* certain particular attributes, he is not consistent with this general definition. In referring to what he calls the sophism of Buridan's ass, in which the unhappy animal remains undecided in his choice, because the two bundles of hay before him are exactly alike, he asks, "What is meant by two bundles of hay exactly alike? They must be undistinguishable by sight, smell, touch, and so forth. But are objects exactly similar as regards the senses, therefore exactly similar as regards the will?"† Now the same objects may be exactly similar, or they may be quite different, with regard to their effects on the will, just as they happen to stand with regard to the opinion which is formed of them by the judgment or regulative element of the will. If they be judged, from their similar appearance, to be exactly alike, that is, if there be no reason to think otherwise—however different they may really be—and they have any effect at all on the palate and on the appetite for them, which

* *Metaphysics*, p. 171. † *Prolegomena Logica.* Appendix, p. 301.

constitutes the dynamic or orectic element of the will, then that effect will be exactly alike for each object. If, for instance, a piece of salt and a piece of sugar, as proposed by Mr. Mansel, were not only exactly similar in *appearance*, but were judged to be so in their *nature;* then, if they had any effect or not on the palate and appetite, that is, if they were desirable or otherwise, they would both be *equally* so, and consequently be similar as regards the *will*. But if, on the other hand, while their appearance was exactly alike, I had reason to believe that they were really different, then, however similar they might appear to the eye, they would not be similar as regards the will; for the difference being pointed out, the *appetite* or dynamic element of the will might be gratified by the one and not by the other. "A lump of sugar and a lump of salt," says Mr. Mansel, "may be similar to the eye: are they therefore similar to the palate? Certainly not; but this proves nothing in the present case, for the active element of the will, or *appetite* for the object, must be necessarily dependent on the palate; and taste is a special sense, immediately dependent for its gratification on its object—the sugar or the salt. But it is precisely on account of its known pleasurable effect on the palate that either the salt or the sugar becomes an object of desire, which, in the absence of any other more influential feeling or desire, will, as formerly explained, terminate in a volition to obtain it. It is evident, then, that in the case supposed, the will must be dependent on the sense for the very condition of its exercise; for without the sense there would be no desire for its object, and consequently no volition. What, therefore, are we to think of Mr. Mansel's extraordinary question on this occasion: "If taste is not dependent on another sense, why may not will be independent of all the senses?"* The case is of course similar when objects are presented not to the external senses, but to the mind in the form of ideas; and in every instance they must be desired for the sake of some sense, appetite, or some emotion which they are capable of gratifying, or they would not be desired at all. It is manifest, then, that from ignorance of the real process of an act of volition, and a desire to maintain the entire independence of the will, Mr. Mansel is necessarily driven into the doctrine of a special faculty.

Hutcheson describes the *will* as "the general calm desire of good and aversion to evil, either selfish or publick, as they appear to our reason or reflection," and as distinguished from "the particular passions towards objects immediately presented to some sense. Thus nothing can be more distinct than the general

---

* *Prolegomena Logica.* Appendix, p. 301.

calm desire of private good of any kind, which alone would incline us to pursue whatever objects were apprehended as the means of good, and the particular selfish passions, such as ambition, covetousness, hunger, lust, revenge, anger, as they arise upon any particular occasion."* Now, although the general calm desire of private good, in the above sense, may, by resulting in action, become the will on that occasion, yet this definition of the will is much too limited in its signification; for to act from a general calm desire of private good is to act from a motive of self-interest; whereas men commonly act in opposition to self-interest, and yet in the most voluntary manner, from a desire to gratify some particular passion or appetite. The covetous man voluntarily and deliberately accumulates his treasure. The hungry man may not only consume food without any view to its beneficial effects, but may indulge his appetite to an extent which he knows to be injurious to his well-being; yet no one will deny that the action is voluntary, except in cases of insanity. The revengeful man may voluntarily and most deliberately pursue his victim, although he knows that he is indulging his passion at the expense of his own interest. It is only when the passions or appetites, and the desires to which they give rise, acquire such intensity as to exclude all deliberation, or overcome any other opposing emotion excited by momentary reflection, and thus hurry their victim along, without the possibility of resistance, that the action ceases to be voluntary. It is true that the calm general desire of private or public good, that is, the desire of personal or social happiness in general, when carried into action, constitutes the highest or most intellectual form of the will, since the very idea of its object, *happiness in general*, is the result of abstract thought, of which even the highest animals are incapable. But the actual enjoyment of happiness, as the object of the desire, depends on the gratification of the passions, affections, and appetites, in some form or other; and the means employed for the attainment of this end consists of voluntary actions which are often the result of much thought and deliberation. One man finds happiness in a moderate indulgence of all his appetites and passions; while another rests his chief enjoyment on the immoderate gratification of one or more, which may be either of a sensual or of an intellectual character. But even self-interest, which gives rise to the calm, general, and prospective desire of private good or happiness, may so engross the mind, and become itself a passion of such intensity, as to exclude even the moderate indulgence of the other passions and the appetites, and thus defeat its own end.†

* Hutcheson *On the Passions and Affections.* Sect. ii.

† See Note A, p. 211.

Reid seemed to consider the *will* as consisting only of reason or judgment in reference to right and wrong, or as corresponding to what some others understand by the moral sense or conscience. "The human constitution," he says, "consists of two parts, the appetites, affections, and passions; and reason. The former constitute the brute part of our nature; the latter, our manly part." Whether this ought to be called reason, or, as some philosophers think, an internal sense or taste, he does not stop to inquire. "By this cool principle," he continues, "we judge what ends are most worthy to be pursued, how far every appetite or passion may be indulged, and when it ought to be resisted. . . . . All wisdom and virtue consists in following its dictates; all vice and folly in disobeying them."* Now, it has already been shown that reason or judgment is only one element—the regulative element—of the will, and therefore alone cannot excite a true act of volition; and even if this "cool principle" be compounded of right reason with some form of feeling or emotion, it can constitute only one form of the will, namely, the *moral* will. If volition were restricted within these narrow limits, it is manifest that in the highest animals every action would be *in*voluntary; and that even in man, not only all those actions which have no kind of *ethical* relation, but all those that are not virtuous or proper—that are not acts of this moral will, or cool principle of right reason—would likewise be *in*voluntary; so that *vice* would have no actual existence, for, like virtue, it can exist only by reason of its voluntary character. But a large majority of our voluntary acts have no relation whatever to morality; and we know from experience not only that those which have this relation are both good and bad, but that *reason* may be *right*, while the *volition* at the same time may be *wrong*. Since, therefore, every volition is the immediate effect of a desire to act, arising out of some feeling, appetite, or emotion, reflected through the intellect, it follows that the moral quality of actions will depend upon whether or not the *desires* which produce them *coincide with*, and *conform to*, the dictates of right reason, or be excited by it. One man will desire to act, and if he be not susceptible of some more influential feeling indicated by right reason, *will* act voluntarily for the gratification of some low animal appetite; while another, with similar temptations, will act from a stronger love and desire of justice, honour, benevolence, a more general love of virtue, or from the still more exalted feeling of love and reverence for the Supreme Being—a feeling which we are commanded to cultivate and cherish with all our hearts and with all our souls. So far, then, do the affections

* Reid: *Active Powers.* Essay ii., chap. ii.

and passions, and the desires to which they give rise, constitute the brute part of our nature, that amongst them we find the source of our noblest and most exalted volitions. "It has long," says Mr. Mansel, "been established as a canon in morals by the soundest writers on the question, that virtue and vice depend, not on the existence of desires, but on their relation to the will."* Here it is evidently implied that the will is a faculty altogether distinct from the desires; whereas I have shown, with sufficient clearness, I hope, that every volition is the immediate effect of a desire to act, under the guidance of reason, for the sake of some appetite or emotion, which is, therefore, the cause of the desire; so that virtue and vice depend, not on the relation of the desires *to the will*, considered as a distinct faculty, but *to right reason* or *moral knowledge*. For without the knowledge of right and wrong there can be no imputation of either virtue or vice, although the actions performed may be quite voluntary, or done with a deliberate intention. On the other hand, there may be a perfect knowledge of right, not only with an entire absence of any desire to act in conformity to its suggestions, but with an opposite desire resulting in an opposite volition; for although scarcely any one can be found to act from an abstract love of vice, or for its own sake, yet from a love or impulse toward some particular object or end, one may desire to act, and may act, in a way which he knows to be contrary to the dictates of right reason, injurious to himself and society, and which on that very account is forbidden under the name of *vice*.

"Desires," says Mr. Mansel, "are not under our control. . . . . We cannot help feeling attracted to pleasant objects; but we can help yielding to the attraction of desire when felt; and we can help putting ourselves in the way of feeling it. . . . . I may give my attention to thoughts calculated to excite desire, and the attention is a voluntary act."† But how do we "help yielding to the attraction of a desire?" Simply by "giving our attention to thoughts calculated to excite," not *that* desire, but some *other* which is capable of controlling or subduing it; and this very act of voluntary attention to such thoughts is itself, as I before stated, the *immediate effect of a desire* to accomplish the end in view. So that all our voluntary control over the attractions or tendencies to action of one desire, consists in opposing it by some other desire which is excited through the medium of reflection. Turn the question in every possible way—view it in every possible light—a complete analysis of the facts of volition, and a legitimate process of reasoning from these facts,

---

* *Metaphysics*, p. 172.

† Mansel: *Metaphysics*, p. 172. This passage is very nearly a translation of one in Cousin's *Fragmens Philosophiques*. 1826. p. 36.

will be found to result in this inevitable conclusion. Nothing but the *à priori* assumption of a special faculty of will, considered as independent of the desires and the understanding, could lead, by any legitimate argument, to an opposite opinion.

It is surprising to find that a very distinguished French philosopher now living, upon whom Mr. Mansel seems to rest for support, has involved himself in still greater contradictions in his exposition of the nature of the Will.* "The free-will," argues M. Cousin, "cannot consist of sensation, for that is purely passive and independent of myself as a cause; neither can it consist of intelligence, for intelligence is not free; it cannot judge otherwise than it does; for example, it cannot judge otherwise than that two and two make four. It must, therefore, obey laws that it has not itself made; and is consequently marked by the same necessity as sensation. If free-will, then, is not to be found either in sensation or intelligence, where," asks M. Cousin, "can we find it, except in the active element or activity?"† Without stopping at present to expose the absurdities involved in these statements, let us proceed to M. Cousin's description of volition. "When the understanding," he continues, "has judged that this or that ought to be done, from such and such motives, I then proceed to act; and first of all, to resolve and say to myself, 'I not only ought to do this—I will do it.' But the faculty which says 'I ought to do it,' is not and cannot be the faculty which says 'I will do it,' 'I resolve to do it.' Here ends the office of the understanding. 'I ought to do it,' is a judgment; 'I will do it,' is not a judgment. Here is a new element which must not be confounded with the preceding: this element is the Will."‡ Now, that a faculty which is not endowed with the attribute of intelligence can resolve, or make a resolution to perform a particular act, and state to itself the proposition "I will do it," is quite impossible—it implies a contradiction; for these operations necessarily involve a process of thought or reflection; and yet in the same passage we are

---

* An analysis and criticism of the different opinions of writers on the nature and acts of the will, will present this difficult subject in different lights, or under a variety of circumstances, and enable me at the same time to answer any specious or subtle arguments that might appear as objections to my own exposition of the nature of volition. It is only by such a course that we can, if possible, put a stop to endless discussions.

† *Cours de l'Histoire de la Philosophie Moderne.* Deuxième Série. 25[ieme] Leçon, tom. iii.

‡ "Quand l'intelligence a jugé qu'il faut faire ceci ou cela, sur tels ou tels motifs, il reste à passer à l'action, et d'abord à se resoudre, à prendre son parti, à se dire à soi-même, non plus je dois faire, mais je veux faire. Mais la faculté qui dit je dois faire, n'est pas, et ne peut pas être la faculté qui dit je veux faire, je prends la resolution de faire. Ici cesse le rôle de l'intelligence. Je dois faire est un jugement; je veux faire n'est point un jugement. Voilà un élément nouveau qu'il ne faut pas confondre avec le precedent; cet élément, c'est la volonté."—*Op. cit.* pp. 349-50.

informed that this assumed faculty is altogether wanting in intelligence, and on that account is the free-will. But, mark again the inconsistency of the passage last quoted with the one that precedes it. What the understanding, we are told, judges expedient to be done, that and that only the free-will determines to do—that is, in every case it depends for the necessary condition of its exercise, as free-will, on the judgment and guidance of the understanding, without which, evidently, it either could not act at all, or must act blindly, without an object, end, or design; and then, as I have just stated, it would be incapable of conceiving a resolution, or forming such a notion as "I will do it." And further, M. Cousin found that the judgments of the understanding are bound by the laws of necessity, over which of course the will can have no control, and on account of which the claims of the understanding to be the faculty of will were at once rejected. But since we have just seen that the "will" is dependent on the judgment for its choice of action, and consequently for its very exercise as will, it follows that it must be dependent on, or subject to, its necessary laws. Upon the ground, therefore, of entire independence, to which M. Cousin points as the criterion of freedom, the active principle can have no more claim than the understanding to the title of free-will. Moreover, since, according to M. Cousin, this active principle, or supposed free-will, is neither sensation nor intelligence, it must necessarily be either appetite or emotion ; for these are the complemental faculties of our conscious existence. But both of them are rejected by M. Cousin, as passive and obedient to laws.

"The phenomena of the will," says M. Cousin, in another work, "present us with the three following steps:—1. A predetermination to perform an act; 2. Deliberation; 3. Choice, or resolution. On consideration," he continues, "it will be seen that reason constitutes the first process entirely, and even the second, for it is reason which also deliberates ; but it is not reason which resolves and determines itself."* Now let us examine these three operations a little more closely, and see whether the above statements be correct. 1. When some desirable object happens to present itself to my senses, or occur to my mind, I naturally feel a desire to obtain it; and, provided there be no sufficient reason and corresponding impulse to the contrary, or other obstacle in the way, my desire will just as naturally spend its force in the *action* necessary to obtain it. If, however, the steps necessary to be taken are not imme-

---

* "Le phénomène de la volonté présente les momens suivans: 1° Prédéterminer un acte à faire. 2e Délibérer. 3° Choisir. Si l'on y prend garde, c'est la raison qui constitue le premier tout entier, et même le second, car c'est elle aussi qui délibère, mais ce n'est pas elle qui résoude et se détermine."—*Fragmens Philosophiques*, Préface, p. xxvii.

diately perceived, or cannot be immediately pursued, the act of course must be delayed for a time; but my desire still remaining ungratified, and therefore striving for the means of gratification —intent on the end and awaiting only its opportunity—is now called a *predetermination* or intention to act, with that end in view. 2. To obtain a knowledge of the necessary means, the same desire of the end and intention stimulate my reason, and co-ordinate my thoughts in a process of deliberation on the circumstances of the case. 3. When this latter process is concluded, and my judgment had decided on the means necessary to be taken, the same desire for the end or the intention fixes upon these means, and having no further obstacle to restrain its impulsive tendency, is spent at once in the voluntary and predetermined *act*. Such, if I mistake not, is the true analysis and explanation of this complex process of volition accompanied by deliberation. The purpose of deliberation will of course differ in different cases, according as the point to be determined be—whether the action shall be performed at all, or what means are necessary for performing it; but in all cases the nature of the process is the same. Now, in each of the three processes above explained, we perceive that both the *active* and *regulative* elements of volition are engaged; for, 1, the predetermination to act is an absolute desire and intention to act with a conscious end or design; 2, the act of deliberation is a special co-ordination of ideas excited by the desire to ascertain the necessary means, and is as much a *volition* as is the special co-ordination of muscles excited by the same desire to carry those means into execution for the predetermined end—which constitutes the third of these processes. It cannot therefore be, as M. Cousin asserts, that *reason alone* performs the first and second of these operations, and that the so-called will or *active principle* is the *sole element* engaged in the third. But let us accompany M. Cousin to his conclusion. "The intelligence or reason," he continues, "which here combines with the will, combines with it in a reflected form: to conceive an end, to deliberate, conveys or implies the idea of reflection. Reflection, then, is the condition of every voluntary act."* This is equivalent to the assertion, that the will, notwithstanding the entire independence assigned to it, cannot act without reflection; or that no action which is not accompanied by reflection can be voluntary; and therefore, that no faculty that can act without reflection can of itself constitute the will, a proposition which is in direct contradiction to M. Cousin's doctrine.

* "La raison qui se mêle ici à la volonté, s'y mêle sous une forme réfléchie; concevoir un but, délibérer emporte l'idée de la réflexion. La réflexion est donc la condition de tout acte volontaire."—*Fragmens Philosophiques,* Préface, p. xxvii.

But, says M. Cousin, "to will means, that, while knowing that I can resolve and act, I deliberate whether I shall resolve —whether I shall act in such or such a way, and choose in favour of one or the other. The result of this choice—of this decision preceded by deliberation and predetermination, is volition —the immediate effect of personal activity. But before I could resolve in this way, I must have known that I could resolve and act—and must have previously resolved and acted in a different manner, without deliberation or predetermination, that is to say, without reflection. The operation which precedes reflection is spontaneity."* The meaning of this passage is simply —that before I can act with reflection, that is, with a conscious design, I must have been conscious of the operation of some unintelligent and blind impulse. But the act of a blind impulse cannot be regarded as my act, since it lacks the co-operation of that intelligence which constitutes the essential and distinctive element of my nature and personal consciousness. What, then, can be the nature of this mysterious faculty of will? It is spontaneity, we are told. But spontaneity is not a faculty—it is merely a *mode of action* of a faculty; to mistake the one for the other, is to mistake an abstraction for a reality. And since this faculty is not intelligence, it must consequently be one or other of the complemental faculties of our conscious being— namely, a sensation, an appetite, or an emotion—all of which, however, are rejected by M. Cousin as having nothing whatever to do with the will; nay more, he maintains that our personal being, which is the will itself, and whose freedom is spontaneity, so far from being *constituted*, is actually *destroyed*, by the sensations, desires, and passions; so that, according to him, whenever we feel, desire, or experience any of the affections, our personal being is at an end!† Thus does M. Cousin fail to perceive that our conscious personality or integrity is constituted, on the one hand, of sensations, desires, and emotions, and on the other hand, of conscious intelligence; and that since the will is our personality, and volition is a conscious act, it must be compounded of both the æsthetic or active, and the intellectual or regulative elements of our nature.

---

* "Vouloir c'est, sachant qu'on peut se résoudre et agir, délibérer si on se résoudra, si on agira de telle ou telle manière, et choisir en faveur de l'une ou de l'autre. Le résultat de ce choix, de cette décision précédée de délibération et de prédétermination est la volonté, effet immédiat de l'activité personelle. Mais pour se résoudre et agir ainsi, il failloit savoir qu'on pouvait se résoudre et agir; il failloit antérieurement s'être résolu, avoir agi autrement, sans délibération, ni prédétermination, c'est à dire, sans réflexion. L'opération antérieure à la réflexion est la spontanéité."

† "La volonté est donc l'être de la personne. Les mouvemens de la sensibilité, les désirs, les passions, loin de constituer la personalité, la détruisent."—*Fragmens Philosophiques*, Préface, xxvi.

But the examples which M. Cousin adduces in support of his position are examples which betray its unsoundness. It is unquestionable, he observes, that we often act without deliberation, by an impulse which is not foreign or external, but spontaneous, and a kind of sudden inspiration superior to, and better than, reflection. Such was the heroic impulse which uttered the "qu'il mourût" of the father of the three Horatii.* But what is the feeling of heroism but an emotion—one of a class of feelings which M. Cousin excludes from all participation in volition.

One more difficulty for M. Cousin, and for all who stand on the same ground. "As the proper character of a spontaneous act," says he, "consists in its not being voluntary, the spontaneous act is not repeated at will, and either passes unperceived, or is irrevocable: it can only ultimately be recalled on condition of its being reflected (through the intellect), that is to say, on condition of its being destroyed as a spontaneous fact."† But according to M. Cousin's previous statement, spontaneity is just that peculiar and essential attribute which gives freedom and independence to the will, and which thus distinguishes it alike from the understanding, the appetites, and the desires. It follows, therefore, as a logical consequence, that the will must be at once spontaneous and not spontaneous—free and not free!‡

That spontaneous (instinctive) movements are the means by which we originally acquire voluntary control over our muscular actions, is, as we have already seen,§ the doctrine of Hartley, Priestley, Bain, and some others, who deny the existence of a special faculty of will. But then this voluntary control

---

* *Fragmens Philosophiques,* p. xxviii. The expression "qu'il mourût," to which Cousin here refers, is to be found in P. Corneille's *Horace*, act iii., sc. vi., which contains some of the finest expressions of heroic grandeur in this or any other poet of ancient or modern times. When Julie comes and announces to the father, Horatius, that two of his sons have been killed in the combat, and that the third, who fought valiantly while his brothers were alive, fled on finding himself opposed and surrounded by three adversaries, the old Roman, instead of deploring, with his daughter Camille, the loss of the two sons who had so gloriously fallen, is filled with a sense of the indelible disgrace stamped on his name by the cowardly and shameful flight of the third; and when his daughter curtly inquired—

"Que vouliez-vous qu'il fît contre trois?"————

————————————"Qu'il mourût,"

was abruptly thundered forth by the heroic and indignant Roman.

† "Le caractère propre d'un acte spontané étant de n'être pas volontaire, l'acte spontané ne se répète point à volonté, et passe, ou inaperçu ou irrevocable, et ne peut être ultérieurement rappelé qu'à la condition d'être réfléchie, c'est à dire, d'être détruit comme fait spontané."—*Fragmens Philosophiques,* Préface, p. xxix.

‡ See Note C, p. 214.

§ See No. VIII. of this Journal, 1862, pp. 586–7.

is possible only under the guidance of intelligence. All the spontaneous movements of the new-born infant are the immediate effects of certain feelings of impulse or inclination, which are only the conscious accompaniments of vital or organic actions taking place in some of the nervous centres. Amongst these spontaneous feelings is the special appetite of hunger, which subsequently excites a desire and volition with a view to its gratification. But does the appetite, which is in this case the active element of the will itself, arise spontaneously, that is, independently within the mind? Or is it dependent on something else? Not on the external senses, but, nevertheless, on a certain state of the stomach, which is itself dependent on external conditions. It is the stomach really that hungers, and calls on the brain, by exciting an appetite, to provide the means of supplying its necessary wants. But its necessary wants are the wants of the entire organism, and consist of certain excitants or substances which we call food, upon which, therefore, the spontaneous action of all the organs of the body, including the brain, with its special centre of appetite, is immediately dependent. How, then, can spontaneity, on the ground of its *perfect freedom and entire independence* as a self-moving power, constitute the free will? "A desire," say Mr. Mansel and M. Cousin, "cannot be free, because we cannot help feeling a desire for what attracts." But can we help feeling the appetite of hunger which arises spontaneously when our stomachs are empty, but which, nevertheless, excites a desire that results directly in volition, whenever we act with a view to its gratification, and which, therefore, on such occasions is the active element of the will? Hence, the so-called spontaneous feelings or impulses, resulting in spontaneous movements, being themselves the result of organic actions of the nervous centres, which in their turn are dependent on external conditions, must also "obey laws which they have not themselves made," and be consequently "marked by the same necessity" as intelligence, which, however, is the only element that can supply the conditions of moral freedom, that is, deliberation and choice.* To attribute freedom to a blind impulse which must necessarily act in one and only one particular way, whether for good or for evil, is as absurd as it would be to attribute freedom to the sun because it emits its rays spontaneously, that is, without external compulsion, and by virtue of its physical constitution. And to reduce the free actions of men to the same level with the unalterable or necessarily invariable operations of mere physical forces, is just the dangerous error which the doctrine of these

---

* See above, p. 200.

writers is intended to oppose, but into which, as we have seen, it must necessarily betray its supporters.

It has been already observed that volition and moral freedom imply spontaneity, as one of its necessary conditions; but to overlook the distinction between spontaneity and will is to confound physical with moral liberty, as M. Cousin and others unwittingly do. Perhaps no writer who has fallen into this error has obscured the fallacy and maintained his position by such powerful reasoning and specious argument as the celebrated Dr. Samuel Clarke. I shall, therefore, briefly examine his premises as they appear in different parts of his works, and deal with him as I have done with Cousin; a task which I consider the more necessary, because he has succeeded in misleading other writers, and amongst these, no less distinguished a philosopher than Dugald Stewart, who considers that his discussions on the subject contain "the most important, as well as powerful, of all his metaphysical arguments. Almost every page in the subsequent history of this controversy," he continues, "may be regarded as an additional illustration of the soundness of Clarke's reasonings, and of the sagacity with which he anticipated the fatal errors likely to ensue from the system which he opposed."* I shall show, however, that the system with its fatal errors which Clarke fancied that he was refuting, are precisely those which must follow necessarily as logical consequences of his own statements, from which, had he perceived those consequences, he would of course have shrunk with horror and disgust.

This great and excellent man, from his zeal to prove the entire independence of the will, contended that it is endowed with a power of *beginning motion*, or a self-moving principle of action, which is independent of everything but the will of the Creator. In a certain sense it is true that man, and every other organized being capable of locomotion, possesses a self-moving principle, inasmuch as such a principle is inherent in, and dependent on, the constitution of the organism which he calls himself; but in every conscious movement, it must manifest itself in the form either of sensation, appetite, emotion, or intelligence; and for these manifestations it is dependent on the instrumentality of special material organs. Free-will, however, or moral liberty, we are told, is not dependent on sensation and intelligence, for neither of these "has anything to do with the question of liberty," which consists solely in the power of acting.† Now besides sensation and mind, there is no other

* *Philosophy of the Active and Moral Powers*, Appendix.

† *Remarks upon Collins's Philosophical Inquiry concerning Human Liberty*; appended to the Collection of Papers.

manifestation of a *motor principle*, except the vital movements by which the organic or vegetative functions of the body are carried on. If, therefore, we exclude sensation and mind—which here is understood as comprehending all our conscious faculties—we reduce man to the condition of the lowest animal, or even the lowest plant, and make his free-will to consist of the merely organic or vital power by which the heart beats or the lungs breathe. "But," says Dr. Clarke, in a different part of the same paper, "the mechanical and involuntary motions of the body, such as the pulsation of the heart, and the like, are all necessary, and are none of them actions." . . . . . "The pulsation of the heart is as necessary a motion as that of a clock." How, then, could Clarke reasonably complain of, and endeavour to refute, his opponent—who made the difference between a man and a clock to consist of nothing but sensation and intelligence—when his own statements, pursued to their logical consequences, would have compelled him to acknowledge that he himself made no difference at all between them; since, after excluding all our conscious faculties of sensation and mind, the only remaining principle of motion in man's nature, upon which, according to him, free-will solely depends, consists in the vital or organic force, whose effects or motions he, at the very same time, admits to be necessary motions, and no more the actions of the man than the motions of the clock are the actions of a clock; that is, that none of them proceed from a self-moving power. Hence, according to Clarke's argument, man has a self-moving power, and has *not* a self-moving power; his own actions are *not* his own actions, and his free-will is *not* free-will.

In other passages this writer calls the "self-moving power" physical liberty or spontaneity. "In beasts," he says, "the same physical liberty or self-moving power is wholly separate from a sense, or consciousness, or capacity of judging of moral good and evil, and is vulgarly called spontaneity." . . . . "In man, this physical liberty is joined with a sense, or consciousness, of moral good and evil, and is therefore eminently called liberty." Now, since there can be no sense or consciousness of moral good and evil without intelligence, it follows that what is eminently called liberty in man, that is, his freedom of will, must be dependent on intelligence as one of its necessary conditions; for, if man can have free-will without intelligence, as Clarke asserts that he can, then his freedom must be the mere physical freedom of the vital force, which Clarke, as we have already seen, declares to be no freedom at all. "The actions of every living creature," we are told, "are all of them essentially free," whether or not they be possessed of intelligence and sensation. By freedom is here meant the capacity of action by the exertion of some inherent force; and by action is meant not the internal vital movements, like those of the heart, but external movements or locomotion. Every plant, therefore, capable of the least spontaneous movement of any of its external parts, must, according to Clarke's argument, be accounted a free agent.

In answer to the question, "whether we are at liberty to will or not to will?" he says, that so far as the understanding is concerned we are not, for the understanding is passive; and so far he is correct, for the understanding alone has no voluntary motor power. But when he says that by virtue of the active faculty, or spontaneity alone, we are at liberty to will or not, he is certainly in error, and states an absurd proposition; for spontaneity, consisting, according to him and others, of something distinct from, and independent of, our conscious faculties of mind and feeling, can, as already stated, be nothing else than the unconscious organic force, and therefore can have no choice, which is the essence of free-will. To the question, "whether we are at liberty to will or choose one or the other of two or more objects?" the answer, he says, is the same—that by virtue of the understanding we cannot; but by virtue of the active faculty, or spontaneity, we can. Here, again, we find the same fallacy and contradiction. The understanding by itself, can only compare one object with another, and judge of their qualities, but possesses no craving or appetite for either. On the other hand, the active principle alone would be unable to form any judgment of the comparative value of objects, and therefore could have no power of choice of one rather than another, but must necessarily crave after, and, in the absence of any obstacle, be attracted towards that to which its properties have a natural tendency; and in this mere craving or attraction for a particular object presented, without the privilege of judging of its comparative value, and of exciting some other kind of feeling or craving, we should be no more at liberty to will or choose one or the other of two or more objects, than a stone thrown into the air would be at liberty to choose whether it should gravitate toward the earth or toward the moon. What Clarke appears to have meant by "choice or will," is the mere exertion of force in action, and towards some particular object; but this alone, in any living being, is no more will or choice than it would be will or choice in a stone, when simply let loose in the air, to gravitate towards the earth instead of remaining stationary.*

It is really surprising that men like Dr. Samuel Clarke,

---

* See Note B., p. 212.

Cousin, Dugald Stewart, and others equally distinguished for their depth of thought and powers of reasoning, could allow themselves to be betrayed into so much perplexity and contradiction. Such, however, is the fact, if we are to place any reliance at all on accurate analysis and legitimate deduction. But how is this fact to be explained? Chiefly, I think, by the influence of a well-meant but mistaken religious prejudice, which biassed the judgment and diverted the attention from the true path of inquiry; so that instead of starting from an accurate analysis of different acts of volition, they began synthetically by assuming the existence of some simple, mysterious, and lawless principle, of which they could form no clear or definite conception, and to which, therefore, as occasion required, they attributed *ad libitum* certain mysterious functions which served to explain, to their own satisfaction, whatever was otherwise inexplicable!

When we reflect on the fact that the doctrines which I have been opposing, have been espoused and defended for centuries by many of the most eminent philosophers, and that in a more or less modified form they still continue to maintain their ground, we might infer, what has proved to be the case, that they are founded on a mixture of truth and error, which are so intimately and peculiarly blended together as to escape distinction by minds which are biassed by particular prejudices. It appears, therefore, that the only chance left of effecting a uniformity of opinion, and stopping so protracted a discussion, lies in a complete examination of every fact and every proposition that can be employed in the premises of this celebrated controversy.

There are certain propositions that seem to be at once so evident and consistent with our own personal experience, that scarcely any one refuses to admit them without examination, or, unless for some especial purpose, thinks of inquiring whether they be absolutely, or only partially, true; while in reality they may be true only in one sense, and false in another, although the truth may happen to be so prominent and glaring as to blind one's perception of the error. Such would appear to be the general proposition that "man and every animal are endowed with a self-moving faculty," or "a power of beginning motion." Now in one sense this expression means, and is intended to mean, that unless he be prevented by external force, a man has the power either to remain at rest, or to begin motion whenever he feels inclined; and in this sense, and so far, the proposition is perfectly true; for if the *motor power* be a feeling, or impulse, which is either suggested or sanctioned by his intelligence, as already explained, he performs an act of volition resulting from the co-operation of those faculties which together, and only together, constitute his conscious self as a personal being. But this is not the sense in which the expression "power of beginning motion" is employed by Clarke and many others; for they mean by it, as already shown, a power which is independent not only of the feelings and the understanding, but of every condition and circumstance except the will of the Creator who bestowed it. This faculty, therefore, they regard as one of the faculties peculiar to the soul. But grant it to be a faculty of the soul; then, since it is said to be neither feeling nor intelligence, it must be not only a blind, but an *unconscious*, faculty, which we can know only by its effects: it cannot, therefore, constitute the will, for volition *implies consciousness.* We know that in different states of profound unconsciousness, or insensibility, occasional spontaneous movements—spasmodic or otherwise—may occur; but no one considers such movements voluntary, or actions of the man himself. And again, "the actions of every living being," says Clarke, "are all of them essentially free." "Liberty," says Dr. Price, "is common to all animals, as well as to all reasonable beings; every animal, as such, possessing powers of self-motion, or spontaneity." If, therefore, activity or spontaneity of action belong only to the soul as a separate immaterial entity, then we must not only suppose a similar entity in some of the lowest animals and plants, because they possess spontaneity, but must deny it in others that are higher in the scale, because the same power is wanting. It is not my intention here to deny the existence, or discuss the question, of a separate immaterial soul, but simply to prove that spontaneity may occur without it, and have its source in the material organism. For it is contended by those who hold the opposite doctrine, that matter, and therefore the material organism, cannot possibly have the power of action, or spontaneity, because it is purely passive, and no agent at all; and for the same reason the same predication is extended to sensation, to the desires, and the emotions. Now, by reflecting on the phenomena of nature, we shall find that there is no such thing as *pure passivity* in matter. Every particle of matter possesses certain properties, that is, certain inherent forces which, in some form or other, are incessantly exerting themselves, or acting upon the forces of other particles. When a body is moved by a foreign or external impulse, resulting from the will of a living agent, it is not purely passive, but resists that impulse, not of course with design, but by reason of the force which it exerts in an opposite direction on surrounding bodies. And the same resistance is offered whenever one particle of matter is moved by another, not from external impulse, but by virtue of its inherent force. So that the supposed passivity and inaction of matter does not arise from any absence of force or power of acting, but simply from the fact that that force or power, under existing conditions, is opposed and restrained by another of superior energy. Every particle of matter, therefore, possesses an inherent principle, or *capacity*, of self motion ; but whether this principle shall spontaneously result in *actual* motion or not, will depend solely on the external and merely accidental conditions of the absence or presence of resistance and restraint opposed to it by other forces.* Now, there is reason for believing that these external conditions may be so regulated and provided as to allow physical forces, or the principle of motion inherent in matter, not only to carry on those *continuous* operations which constitute life in an organism, but even to *originate* at intervals the *occasional* phenomena of outward action and locomotion, as I shall take a future opportunity of explaining.

In reviewing these inquiries, then, on the nature of the will, we find, not only that the theory which I have adopted and founded on a careful analysis of different forms of volition is consistent with itself and competent to afford a satisfactory explanation of the facts to which it may be applied, but that every opposite theory that has been proposed, when properly examined and tried by a similar test, has proved to be wholly untenable, and, when followed out to its logical consequences, has resulted in absurdity and contradiction.

It cannot fail to be perceived, that the facts elicited and the conclusions drawn in the course of this discussion must bring into clearer light many at least of the obscurities that belong to the dark and difficult question of moral liberty. For the consideration of this subject, however, the urgency of my anatomical and physiological investigations will not at present allow me the requisite leisure.

---

NOTE A., p. 197.

Bishop Butler has some excellent remarks on this tendency of immoderate self-love and self-interest. "Happiness or satisfaction," says this sturdy philosopher, "consists only in the enjoyment of those objects which are by nature suited to our several particular appetites, passions, and affections. So that if self-love wholly engrosses us, and leaves no room for any other principle, there can be absolutely no such thing at all as happiness or enjoyment of any kind whatever; since happiness consists in the gratification of particular passions, which sur-

* See Note D, p. 218.

passes the having of them . . . . . Our fondness for a child is not generally thought to be for its advantage; and if there be any guess to be made from appearances, surely that character we call selfish is not the most promising for happiness. Such a temper may plainly be, and exert itself in a degree and manner which may give unnecessary and useless solicitude and anxiety, in a degree and manner which may prevent obtaining the means and materials of enjoyment, as well as the making use of them. Immoderate self-love does very ill consult its own interest; and how much soever a paradox it may appear, it is certainly true, that, even from self-love, we should endeavour to get over all inordinate regard to, and consideration of, ourselves."—*Butler's Fifteen Sermons.* Sermon XI.

NOTE B., p. 208.

Dr. Samuel Clarke, in one of his controversies on this subject, conducted his argument in a manner so specious as to silence completely, if he did not perfectly convince, his opponent.* Nevertheless, through the whole train of reasoning there runs a fallacy which, on account of the importance of the subject to which it relates, I shall not hesitate to expose. It will be seen that Clarke's fundamental error lies in his confounding volition with mere spontaneity of action, and mere physical with moral liberty; together with his notion of what constitutes an agent. "So far," he says, "as anything is passive, so far 'tis subject to necessity; so far as 'tis an agent, so far 'tis free; for action and freedom are, I think, perfectly identical ideas." Between the final perception of the understanding, which is passive, and the first operation or exertion of the active faculty or self-motive power, there is not, he says, any connexion at all.† Now, it is quite true that there is no unconditional or absolutely necessary connexion between these two processes; for there may be an exercise of mind without motor action, and motor action without exercise of mind; but then, as we have already seen, neither the one nor the other alone can constitute an act of volition; so that, although action, absolutely, may be perfectly independent of mind, still, whenever that action is to be accounted *voluntary*, or performed with an object in view, "the perception of the understanding, which is passive, and the exertion of the active or self-moving power," must, under these conditions, be *necessarily* connected. But Clarke maintains that, "in their not being connected lies the difference between action and passion; which difference is the essence of liberty." From

* See his correspondence with a gentleman of the University of Cambridge, in the Appendix to his Correspondence to Leibnitz.

† Answer to First Letter.

which it must be inferred, that whenever the spontaneous self-motive faculty acts in conjunction with mind, it is passive—is a slave, and no agent at all! And yet, further on, he declares that "the self-motive power is (in all animals) spontaneity, and (in rational ones) what we call liberty;" so that if it be liberty only in rational animals, that liberty must be dependent on their reason; an inference which is in direct contradiction to his previous statements, as well as to others that occur elsewhere. "A man's understanding," he continues, "judges of what he is to do as his eyes discern the way; but a blind or winking man has power to walk without seeing, and every living agent has a physical power to act, whether he makes any use of his judgment and understanding or no. Unintelligent matter," he adds, "can be no agent, because action supposes (in every notion of it) life and consciousness; but that consciousness which makes action be action is entirely a distinct thing from that perception or judgment by which a man determines beforehand concerning the reasonableness or fitness of what he is about to act. An agent overruled by a blind impulse is a contradiction in terms; for then he is not at all an agent, but a mere patient. But an agent acting not according to the last judgment of his understanding, is like a man shutting his eyes and walking at a venture down a precipice."* The specious sophistry of this passage appears to have been that which especially staggered and silenced Clarke's opponent. But, setting aside some errors in it, which are exposed in criticisms on Cousin,† let us examine the statements contained in the four last passages. Well, then—he compares the light or judgment of the understanding to the light or discerning faculty of the eye; and asserts, substantially, that as a man physically or organically blind has the power to walk, although he cannot see where he is going, so a living agent, intellectually blind, has the power to act, although he does not understand what he is doing. Now these two cases are not at all parallel, and can be compared only in a figurative sense. It is quite true that a man organically blind has the power to walk or act without seeing. It is equally true that a living agent has the power to act without employing his understanding; but there is this essential and important difference between the two cases,—that the blind man, although deprived of the use of his eyes, employs his understanding, and has, therefore, not only the power to walk, but the privilege of judging whether it be advisable or prudent to walk without the guidance of his eyes—whether, in fact, he will not probably walk into danger, and whether he had not better remain still;

* Ibid. Answer to Third Letter. † See p. 203.

whereas a living agent without understanding, or one acting without in any way employing his understanding (which is practically the same thing), does not judge and consider the circumstances and possible consequences of the action, and has, therefore, no privilege of choosing any other course, nor any reason within himself for restraining the impulse to action which urges him on, perhaps, to utter but unforeseen destruction, and which therefore is, in every sense, *a blind impulse overruling him.* But, according to Clarke's statement, "an agent overruled by a blind impulse is a contradiction in terms; for then he is not at all an agent," but merely passive. It follows, therefore, that a living being acting without understanding is not an agent at all—that is, he is not a free being; for "action and freedom," we are told, "are perfectly identical ideas." But this necessary conclusion is in flat contradiction to the statements of Clarke, and the points for which he is contending. In the last sentence of the same passage he varies the two cases a little by comparing "an agent acting not according to the last judgment of his understanding," to "a man shutting his eyes and walking at a venture down a precipice." Here we have again the same fallacious kind of comparison as that which I have just exposed; for, in the first case, the supposed agent, acting without understanding, follows an *intellectually* blind impulse, which urges him on for good or for evil; whereas the man although organically blind, foresees intellectually and understands the danger to which he would expose himself by "walking at a venture down a precipice;" and the dread of this danger, excited by reflection, would induce him not to be so foolish as to shut his eyes; or, if he must shut his eyes, not to risk a trip down the precipice. We see, then, that the statements of Clarke, when pursued to their logical consequences, lead necessarily to the conclusions which they were intended to oppose.

NOTE C., p. 204.

I have thought proper to refute all these statements of M. Cousin, more particularly because they are not peculiar to him, but are continually employed by others in support of the same general conclusions. Nor must it be supposed, because their fallacy is evident by the light in which I have placed them, that there is therefore no difficulty in detecting it as they stand in the author's works; for many of the statements which I have brought closely together and confronted with each other, are scattered widely apart, and moreover supported separately by specious and subtle reasoning, clothed at times in the language of metaphor, which gives, indeed, a charm to the diction, but ambiguity to the meaning.

NOTE D., p. 211.

It is evident, therefore, that the so-called *vis* inertiæ is nothing more than the force which every particle of matter exerts towards every other particle under certain conditions; and is the same force which, under other conditions, manifests the phenomena of activity and self-motion. A body at rest—as on the surface of the earth—will remain at rest, until either it be compelled to move by some external impulse, or its external relations be so altered as to allow its own inherent force to result spontaneously in motion. But why does the body remain at rest or manifest a state of inertia? Not from a want of inherent force, or capacity to move, but simply because the force which it exerts towards the earth's centre happens to be greater than that which it exerts towards other objects; while, at the same time, its tendency to move towards that centre is impeded by the solidity of the earth itself, and consequently it remains at rest. If a stone be suspended, or supported by any means whatever, so soon as the support is withdrawn the stone begins to move towards the earth spontaneously—that is, by its own force, and without the application of any external impulse. It would be futile and absurd to object that the stone is passively attracted by the earth, for we know that the attraction is reciprocal, and in the direct ratio of the respective masses; and even if the attraction were only on the one side, the truth of the proposition, that matter exerts an inherent power of self-motion, would be proved by the fact that the earth itself is matter. It is a law in mechanics, that the inertia of a body is in the direct ratio of its weight; but its weight is its gravitating force. The inertia of material particles, however, arising from gravitation, is being continually overcome, in some way or other, by the interference of other forces with which matter is also endowed, and by which, from their successive action, other new states of inertia and of motion are alternately induced. Such are the attractions and repulsions arising from cohesion, chemical affinity, electricity, magnetism, light, heat; from the forces engaged in the phenomena of evaporation *in vacuo;* of endosmose and exosmose; the equal diffusion of fluids and gases of different densities, and in the latter case, even when the heavier gas is below the other. From the reciprocal interference of all these forces, there arises, in the inorganic world, a succession of changes, as varied and uninterrupted, and as conspicuous for their order and marvellous design, as are the changes that occur in the living organism.

It was believed by the ancient philosophers that in all things there is both an active and a passive principle. The passive principle is matter: the active principle is the Divine Reason which resides within it.* The elements consist of both these principles together, and therefore are both active and passive. This matter, however, is not the matter which we see, and feel, and handle; but a certain *materia prima* (ὕλη πρωτή), which consists of a substance (ουσια) void of all properties, but yet the common or primary substratum (τὸ πρῶτον ὑποκείμενον) of them all. It is therefore without body, or bulk, or form (ἀσώματος, αμεγεθης, αμορφος). It is neither earth, air, fire, nor water, nor any of the things they produce or are produced from; but is a certain invisible and formless kind of existence, which, however, is the recipient of all things (ἀνόρατον εἶδός τὶ καὶ αμορφον, πανδεχες. Plato, *Timæus.* See also Aristotle, *Physic.*, lib. i. cap. ix. *De Gen. et Corr.*, lib. i. cap. vii., and lib. ii. cap. ix.; and *Metaphys.* lib. xii. cap. iii.) This opinion was founded on the continual flux or transmutation of one element into another. That which we now call water, says Plato, on being converted into solid matter, appears to become stone and earth: on being evaporated it becomes aëriform: air when burned becomes fire, and so on; all things appearing, as in a circle, to afford generation to each other (κύκλον τε οὕτω διαδιδόντα εἰς ἄλληλα, ὡς φαίνεται, τὴν γενέσιν). Who then, he continues, can assert with confidence that any of these is one thing rather than another? Wherefore, it would be safer to say, for instance, that fire is not this or that thing, but such-like—that is to say, something fiery; that water is not this or that, but some such thing—namely, something watery. But that in which each thing appears to be first generated and then destroyed as such, is alone worthy of those names which we give to this thing or the other. For that is always the same, and receives all things without putting on the specific form of any (*Timæus*). This common nature and universal recipient, is the *materia prima* or common substratum —a privation of all properties and forms, but retaining the capacity of assuming all.†

Now, at the present day, the notion commonly entertained of what we call matter is substantially identical with this doctrine of the ancients. We talk of a substratum or substance as distinct from all its properties, which inhere in it. But a supposed substance, distinct from, and capable of existence without properties, is not a substance at all—it is a mere negation of existence—a mental abstraction and not a material reality. We can

* Diogenes Laertius, lib. vii.

† Timæus and Plato seem to have regarded this mysterious substance as identical with extension or space; Timæus calls it τόπος and χώρα; and Plato, χώρα and ἕδρα. The same view of it was taken by Descartes, for the same reasons, but without stating whence he derived it.—*Principia Philosophiæ,* pars prima, sect. liii., and pars secunda, sects. iv. x. and xi.

form a conception of matter only by the effects which these properties produce on our organs of sense, and which we must attribute to certain forces acting in this way. Beyond these we know nothing, and can form no adequate conception of anything else as belonging to matter.* We know that, like the imaginary substratum, they are variously correlated, and capable of manifesting themselves in a variety of ways.† But the forces are not something distinct from their properties, and capable of existence or even conception without them; for each property is merely the mode of action—the *modus operandi* of the forces themselves—the formal law according to which they are unceasingly exerting themselves, in one way or other, whether or not their effects happen to be manifested to our senses, or appear as sensible phenomena.

And, if I may speak reverentially on the subject, the view which I have just propounded appears to be the only one by which the creation of matter can be adequately conceived by the human mind. If in nature there be nothing beyond a system of forces, which are ever energizing or exerting themselves in the form of properties, and by their various effects on our senses, produce in us the sensations and notions of what we call matter—then matter, thus considered, may be conceived as a production or evolution of the Divine mind itself. In man, the mental conceptions, with the feelings or emotions, which are engaged in volitional acts, are constantly resolved, within the material organism, into the *unconscious* nerve-forces, of various kinds, which, in their composition with the similarly-organic forces inherent in, or rather *constituting* some of the organic tissues, result in a variety of actions which carry out the design of the volitional conception out of which they were resolved. Now, since the unconscious nerve-forces, thus evolved, are similar in kind to the other organic forces which constitute organic matter, we may conceive how the conscious conceptions of the

---

* It is the visible and tactile extension and solidity of bodies that suggests the notion of some substratum apart from the subtle properties of matter. But visible and tactile solidity are only the effects of forces; in the one instance producing certain organic changes in our optic nerves and nerve-centres; and, in the other instance, offering resistance to our *own muscular force*. Many bodies having both visible and tactile solidity may be reduced to states in which they lose both, and may be again brought back to their original condition; while others that are unappreciable by our senses in their ordinary state, may, by merely changing their conditions, assume the state of visible and tactile solidity.

† It is not, however, always correct when it is said that one force is *converted* into another. When electricity, for instance, appears from the action of heat on metal, it is not the heat alone, under another form, but the resultant of the composition of the force of heat with the forces which it meets with in the metal. It is equally incorrect when it is said that heat and light are converted into vital force, for vital actions are, in such cases, the resultant of the composition of the forces of heat and light with the forces which they encounter in the organism.

Divine mind, as volitional acts of creation, can be resolved, as it were, in a similar way, into the various unconscious forces which constitute the elements of the material world.* And without speculating on the question, whether the specific forms of matter arose out of the differentiation of other forms of a more general kind, we may conceive it to be probable that these forces, by their laws of reciprocal action on each other in the system of the world, would tend to the material realization of that unity and that design which were involved in the conceptions and volitional acts of the Divine mind out of which they were themselves evolved.

In this idea—or theory, if it may be so called—of creation, there is nothing pantheistic; or if there be, it is certainly less pantheistic than the common notion, that the Divine mind itself is present in every particle of matter, however gross or vile, constituting its essential and efficient properties. For, according to the view which I have propounded, the material forces in nature are no more the *conscious personality* of the Deity than the nerve-forces evolved from acts of human volition are the *conscious personality* of man.

These views, although submitted with deference, are very different from the incomprehensible and baseless dreams of ancient and even modern philosophers on the creation of the material world; for they are based on the revelations of experimental science. And thus it is that the speculative flights of the metaphysician, and the bold assumptions of *à priori* reasoning, can be corrected only through the lapse of ages by the severer lessons of experience and the sober induction of facts which await disclosure at their appointed time. For the truths of natural science—the truths of nature, as apprehended by the human mind — have a correlative mode of production, a correlation of development, *in time.* Facts are slowly collected, which in the course of generations explain, or are explained by others, to which, by the laws and constitution of the mind itself, they successively and necessarily give rise; and thus from age to age, although with unequal course, the tide of discovery flows on, and science becomes the growth of combined experience and thought. Observation, experiment, and induction alternately enlarge or enlighten the field of research, and point out each other's path. The gradual, slow, and often irksome business of experience must precede the discovery of all great truths, which neither the profound thought

* This argument from analogy will be the more satisfactory if, as there is the strongest reason to believe, the nerve-force, and therefore nerve-matter, with the rest of the vital forces, be nothing more than physical forces under peculiar conditions of operation.

nor the subtle sagacity of the philosopher will enable him to realize or reach, except from the high and stable ground which time and labour have raised. A Pythagoras, indeed, by a happy thought, or unaccountable insight, might catch, as it were, at the shadow of a far-distant truth; but the accumulated toil of ages was required to embody, fix, and confirm it: for even Newton himself, with all the strength and with all the light of human genius, would have failed, or probably have failed, to grasp the great fact, had he lived at an earlier period, or at least anterior to the discovery of Kepler's laws, which in his deep and fertile thought at once suggested and were explained by—nay, were identified in abstract form with—those wonderful and beautiful theorems on the ratios of tangential to centripetal forces, in which he might be said to have concentrated the scattered light struck out through many generations by philosophers who never could have anticipated the part they were destined to partake in the discovery of the system of the world!

